# The EXO70 inhibitor Endosidin2 alters plasma membrane protein composition in Arabidopsis roots

**DOI:** 10.3389/fpls.2023.1171957

**Published:** 2023-05-31

**Authors:** Xiaohui Li, Peipei Zhu, Yen-Ju Chen, Lei Huang, Diwen Wang, David T. Newton, Chuan-Chih Hsu, Guang Lin, W. Andy Tao, Christopher J. Staiger, Chunhua Zhang

**Affiliations:** ^1^ Department of Botany and Plant Pathology, Purdue University, West Lafayette, IN, United States; ^2^ Center for Plant Biology, Purdue University, West Lafayette, IN, United States; ^3^ Department of Biochemistry, Purdue University, West Lafayette, IN, United States; ^4^ Department of Chemistry, Purdue University, West Lafayette, IN, United States; ^5^ Department of Statistics, Purdue University, West Lafayette, IN, United States; ^6^ Department of Mathematics, Purdue University, West Lafayette, IN, United States; ^7^ School of Mechanical Engineering, Purdue University, West Lafayette, IN, United States; ^8^ Department of Biological Sciences, Purdue University, West Lafayette, IN, United States

**Keywords:** plasma membrane protein enrichment, exocytosis, proteomics, small molecule inhibitors, exocyst

## Abstract

To sustain normal growth and allow rapid responses to environmental cues, plants alter the plasma membrane protein composition under different conditions presumably by regulation of delivery, stability, and internalization. Exocytosis is a conserved cellular process that delivers proteins and lipids to the plasma membrane or extracellular space in eukaryotes. The octameric exocyst complex contributes to exocytosis by tethering secretory vesicles to the correct site for membrane fusion; however, whether the exocyst complex acts universally for all secretory vesicle cargo or just for specialized subsets used during polarized growth and trafficking is currently unknown. In addition to its role in exocytosis, the exocyst complex is also known to participate in membrane recycling and autophagy. Using a previously identified small molecule inhibitor of the plant exocyst complex subunit EXO70A1, Endosidin2 (ES2), combined with a plasma membrane enrichment method and quantitative proteomic analysis, we examined the composition of plasma membrane proteins in the root of Arabidopsis seedlings, after inhibition of the ES2-targetted exocyst complex, and verified our findings by live imaging of GFP-tagged plasma membrane proteins in root epidermal cells. The abundance of 145 plasma membrane proteins was significantly reduced following short-term ES2 treatments and these likely represent candidate cargo proteins of exocyst-mediated trafficking. Gene Ontology analysis showed that these proteins play diverse functions in cell growth, cell wall biosynthesis, hormone signaling, stress response, membrane transport, and nutrient uptake. Additionally, we quantified the effect of ES2 on the spatial distribution of EXO70A1 with live-cell imaging. Our results indicate that the plant exocyst complex mediates constitutive dynamic transport of subsets of plasma membrane proteins during normal root growth.

## Highlights

• The proteomics profile of Arabidopsis plasma membrane proteins changes following 2-h treatment with the EXO70A1 inhibitor, ES2.

## Introduction

The plant plasma membrane serves as a selective physical barrier between intracellular contents and the environment as well as a signaling platform for transmitting information from the exterior to induce cellular responses. Plasma membrane proteins can be classified as integral, lipid-anchored, and peripheral, based on the mode of association with the plasma membrane (Stillwell, 2016). Integral membrane proteins possess one to several transmembrane domains; lipid-anchored proteins associate with the plasma membrane through covalently attached fatty acids; peripheral membrane proteins associate with the plasma membrane through interactions with other membrane proteins, polar head groups of the lipid bilayer, or both. Several well-characterized plasma membrane-localized nutrient transporters, hormone receptors and transporters, and cell wall biosynthetic enzymes are known to undergo constitutive delivery and turnover (Li et al., 2002; Friml, 2010; Takano et al., 2010; Kleine-Vehn et al., 2011; Irani et al., 2012; Bashline et al., 2013; Chen et al., 2015; Wang et al., 2015; Zhu et al., 2018; Vellosillo et al., 2021). Proteomic analyses of plasma membrane proteins reveal that the composition is altered in response to biotic and abiotic stresses (Nohzadeh Malakshah et al., 2007; Nouri and Komatsu, 2010; Elmore et al., 2012; Takahashi et al., 2013; Nie et al., 2015; Cao et al., 2016; Chen et al., 2016; Voothuluru et al., 2016); however, the mechanisms for delivery of most of these plasma membrane proteins are not well characterized. Investigating how plants maintain the variety, abundance, and dynamics of plasma membrane proteins is essential for understanding cell growth, development, and response to stresses.

The conventional plant secretory pathway involves trafficking through the endoplasmic reticulum, Golgi and post-Golgi vesicles to deliver lipids and proteins to the plasma membrane or extracellular space (Denecke et al., 2012; Drakakaki and Dandekar, 2013; Zhu et al., 2020; Aniento et al., 2022). The exocyst complex is a conserved octameric protein complex that primarily functions to mediate the tethering of secretory vesicles to the site of membrane fusion during the last step of exocytic trafficking (Elias et al., 2003; Zhang et al., 2010; Žárský et al., 2013; Wu and Guo, 2015; Synek et al., 2021; Žárský, 2022). In addition, the exocyst complex also associates with the endocytic and autophagy machinery to modulate membrane recycling and autophagy (Prigent et al., 2003; Kulich et al., 2013; Žárský et al., 2013; Ortega et al., 2022; Žárský, 2022). One plant exocyst subunit, EXO70A1, was recently characterized as a landmark for exocyst complex recruitment to the plasma membrane, through its interactions with anionic phospholipids (Synek et al., 2021). Loss of EXO70A1 function disrupts the recruitment of other exocyst subunits to the plasma membrane in Arabidopsis root epidermal cells (Synek et al., 2021). Some known cargo proteins for plant exocyst complex-mediated trafficking include PIN auxin transporters (Drdová et al., 2013), brassinosteroid receptor (Zhang et al., 2016), and cellulose synthase complexes (Zhu et al., 2018; Zhang et al., 2021). However, it is not known what other plasma membrane proteins are delivered to the plasma membrane through the exocyst complex. Whereas the half-life for turnover of plasma membrane proteins in mammalian cells varies from a few hours to a few days (Tweto and Doyle, 1976; Doyle and Baumann, 1979; Chu and Doyle, 1985), we know much less about the mechanisms and dynamics of protein delivery to the plasma membrane in plants.

Previously, the small molecule Endosidin2 (ES2) was shown to target the EXO70A1 subunit of Arabidopsis exocyst complex and to inhibit exocytosis in plant and mammalian cells (Zhang et al., 2016; Huang et al., 2019). Short-term ES2 treatment inhibits the transport of PIN-FORMED (PIN) family auxin transporter, PIN2, and the brassinosteroid receptor BRI1, to the plasma membrane (Zhang et al., 2016), and also impairs the recycling of PIN2 (Lešková et al., 2020). In mammalian cells, ES2 treatment reduces the cell surface abundance of a membrane-anchored metalloproteinase MT2-MMP (Gómez-Escudero et al., 2017). In addition, given the role of the exocyst complex in autophagy, a recent study examined the consequences of ES2 treatment in autophagy events in mammalian cells, and found that a 24-h ES2 treatment resulted in decreased autophagy (Ortega et al., 2022). To characterize changes in plasma membrane protein composition after short-term inhibition of the exocyst complex, and to identify additional proteins that are transported through the exocyst complex, we used ES2 to inhibit the exocyst complex. We enriched plasma membrane from roots of Arabidopsis seedlings treated with ES2 and DMSO solvent control and identified proteins using mass spectrometry analysis. We then performed quantitative and comparative proteomic analyses to identify plasma membrane proteins with altered abundance in ES2-treated samples and considered proteins with reduced abundance as candidate cargo proteins of exocyst-mediated trafficking in plants. We identified a subset of proteins with significantly reduced abundance at the plasma membrane after two hours of 40 μM ES2 treatment. Gene Ontology analysis of proteins with reduced abundance demonstrated enrichment for functions in cell growth, cell wall biosynthesis, membrane transport, hormone signaling, and stress responses. The abundance of many protein kinases and several uncharacterized proteins was reduced by ES2 treatment as well. Our results show that the exocyst complex mediates dynamic transport of a diverse set of proteins essential for plant growth and environmental adaptation.

## Materials and methods

### Plasma membrane enrichment

*Arabidopsis* PIP2A-GFP seeds (Cutler et al., 2000) were sterilized and sowed on half-strength Murashige and Skoog (½ MS) media with vitamins (Caisson Labs, Smithfield, UT, USA), 1% (w/v) sucrose, and 0.8% (w/v) agar (Fisher Scientific, Waltham, MA, USA). The seeds were well separated from each other in both horizontal and vertical orientations on 10 cm x 10 cm square petri plates. Plates were placed in vertical orientation so that the seedings grew on the surface of the agar media. The plants were grown in a growth chamber (Percival Scientific, Perry, IA, USA) under continuous light of 130 μmol m^-2^s^-1^ at 23°C. Six-day-old seedlings were removed from the agar plates and transferred to liquid ½ MS media with 1% (w/v) sucrose and 40 μM ES2 or 0.5% DMSO (mock). After two hours of treatment under light, the roots of the seedlings were quickly excised and transferred to the plasma membrane enrichment buffer. The process of plasma membrane enrichment followed exactly the published protocol (Collins et al., 2017). To evaluate the enrichment of plasma membrane proteins by Western blot assay, 12 μg of protein from each fraction was loaded onto SDS-PAGE gels and the presence of PIP2A-GFP, Sec12, and SYP41 was detected after transfer to PVDF membrane. The anti-GFP antibody was used at 1:1000 dilution (Takara Bio USA, catalog # 632381, San Jose, CA, USA); anti-Sec12 antibody (Bar-Peled and Raikhel, 1997) was used at 1:5000 dilution; and anti-SYP41 antibody (Bassham et al., 2000) was used at 1:500 dilution. HRP-conjugated goat anti-mouse and goat anti-rabbit antibodies were used at 1:10,000 dilution. The plasma membrane enrichment experiments were repeated four times under identical conditions.

### Methanol-chloroform precipitation

The mass spectrometry sample preparation procedure was based on previous literature (Hsu et al., 2018). Protein concentrations from enriched plasma membrane fractions were quantified using the bicinchoninic acid method. For each sample, 250 μg of enriched plasma membrane protein was processed for mass spectrometry analysis. Proteins from each sample were reduced and alkylated with 10 mM tris-(2-carboxyethyl)phosphine (TECP) and 40 mM chloroacetamide (CAA) at 95°C for 5 min. Methanol, chloroform, and ddH_2_O were added to each 100 µl sample in a volume ratio of 4:1:3, respectively (Wessel and Flügge, 1984). The solution was mixed and centrifuged at 16,000x g for 3 min to obtain aqueous and organic phases. Four volumes of methanol were added to the sample after removing the upper aqueous layer carefully without disturbing the intermediate protein disk. The solution was mixed and centrifuged again at the same settings. The supernatant was removed, the precipitated protein pellet was washed with 400 µL methanol, and the sample was air-dried.

### Protein digestion

The precipitated protein pellet was dissolved with 100 µl of 12 mM sodium deoxycholate (SDC) and 12 mM sodium lauroyl sarcosinate (SLS) in 100 mM Tris-HCl (pH 8.5). Protein amount was quantified by BCA assay (Thermo Fisher Scientific). Dissolved protein extract was diluted 5-fold with 50 mM triethylammonium hydrogen carbonate buffer (TEAB) and digested with Lys-C (Wako Chemicals USA Inc., Richmond, VA, USA) in a ratio of 1:100 (w/w) enzyme-to-protein ratio for 3 h at 37°C, and trypsin (Sigma-Aldrich, St. Louis, MO, USA) in a ratio of 1:50 (w/w) enzyme-to-protein ratio for overnight at 37°C. SDC and SLS were removed by adding 100% acetyl acetate in a ratio of 1:1 (vol/vol) sample to acetyl acetate volume ratio. The digests were desalted by homemade stage tip with styrene divinyl benzene (SDB-XC) membrane (3M) after the digests were acidified with 10% trifluoroacetic acid (TFA) to a pH ~3.

### Dimethyl labeling

Every 50–100 µg of tryptic peptides was dissolved in 100 µl of 100 mM TEAB and mixed with 4 µl of 4% ^13^CD_2_O or ^12^CH_2_O, and then 4 µl of freshly prepared 0.6 M sodium cyanoborohydride was immediately added (Boersema et al., 2009). The mixture was agitated for 1 h at room temperature. The reaction was quenched by adding 16 µl of ice-cold 1% ammonium hydroxide and agitated for 1 min. Heavy and light dimethyl-labeled peptides were mixed after the labeled peptides were acidified with 20 µl of 10% formic acid and then fractionated by basic pH reverse-phase stage tips.

### Basic pH reverse-phase fractionation

Two mg of the Magic C18-AQ beads (5 µm particles) were suspended in 200 µL of methanol and loaded into a 200 µL StageTip with a 20 µm polypropylene frit (Agilent, Santa Clara, CA, USA) (Dimayacyac-Esleta et al., 2015). The C18 StageTip was activated with 50 µL of 100% acetonitrile (ACN) and washed with 50 µL of 200 mM ammonium formate, pH 10. After the StageTip was activated and washed, 25–50 µg of dimethyl-labeled peptides were fractionated from the StageTip with 50 µL of 6 different ACN concentrations: 5, 10, 15, 20, 25% and 80% of ACN in 200 mM ammonium formate, pH 10. The eluted peptides were dried and stored at -20°C.

### LC-MS/MS analysis

Twenty-five µg of dimethyl-labeled peptides used in fractionations were dissolved in 5 µL of 0.3% formic acid (FA) with 3% ACN and 4 µL of each fraction injected into an EasynLC 1000 (Thermo Fisher Scientific). Peptides were separated on a 45 cm in-house packed column (360 µm OD × 75 µm ID) containing C18 resin (2.2 µm, 100Å, Michrom Bioresources, Auburn, CA, USA) with a 30 cm column heater (Analytical Sales and Services, Flanders, NJ, USA) set at 60°C. The mobile phase buffer for 25 µg peptide fractions consisted of 0.1% FA in ultrapure water (buffer A) with an eluting buffer of 0.1% FA in 80% ACN (buffer B) run with a 150 min gradient of 5–30% buffer B at a flow rate of 250 nL/min. The Easy-nLC 1000 was coupled online with a Velos Pro LTQ-Orbitrap mass spectrometer (Thermo Fisher Scientific). The mass spectrometer was operated in the data-dependent mode in which a full MS scan (from m/z 350–1500 with a resolution of 30,000 at m/z 400) was followed by MS/MS of the 10 most intense ions being subjected to collision-induced dissociation (CID) fragmentation (normalized collision energy–30%, automatic gain control–3E4, max injection time–100 ms).

### Proteomics data analyses

The raw files were searched directly against the *Arabidopsis thaliana* protein database (TAIR10) with no redundant entries using MaxQuant software (version 1.6.1.0) (Tyanova et al., 2016a) with 1% false-discovery rate (FDR) cutoff for protein and peptide. The first peptide precursor mass tolerance was set at 20 ppm, and MS/MS tolerance was set at 0.6 Da. Search criteria included a static carbamidomethylation of cysteines and variable modifications of oxidation on methionine residues and acetylation at N-terminus of proteins. The search was performed with full tryptic digestion and allowed a maximum of two missed cleavages on the peptides analyzed from the sequence database. Dimethyl-labeling quantitation was performed by setting the multiplicity as 2 (DimethLys0 and DimethNter0; DimethLys6 and DimethNter6). Re-quantify and match between runs function were enabled.

After database searches, the Perseus software platform (Version 1.6.1.3) (Tyanova et al., 2016b) was used to further analyze the MaxQuant-derived protein groups file. First, the rows marked to be “only identified by site”, “reverse” and “potential contaminants” were filtered out. The Heavy/Light ratio was transformed to Light/Heavy (L/H) ratio for convenience, and then to log_2_L/H ratio. Normalization was performed by median subtraction. Proteins with less than 3 valid values among the 4 replicates were filtered out. One sample t-test was performed with both side and p-value threshold of 0.05. Proteins that passed t-test and with a mean log_2_L/H ratio ≤ -0.32193 were considered to be 20% reduction candidates. Histogram and multi-scatter plots were generated by Perseus software. The reduced abundance candidate list was uploaded to PANTHER platform for Gene Ontology (GO) analysis, including functional classification and statistical overrepresentation (Mi et al., 2013; Mi et al., 2017).

The Venn diagram was generated using the online tool provided by Van de Peer Lab at the Bioinformatics and Evolutionary Genomics Core of Ghent University, Belgium (http://bioinformatics.psb.ugent.be/webtools/Venn/). The presence of signal peptide and transmembrane domains in candidate proteins was predicted using the DeepTMHMM package (v1.0.24) provided by Department of Bio and Health Informatics, Technical University of Denmark (https://dtu.biolib.com/DeepTMHMM) (Hallgren et al., 2022).

### Confocal microscopy

For imaging of fluorescence-tagged protein marker lines, 6-d-old light-grown seedlings expressing PIN2-GFP (Xu and Scheres, 2005), BRI1-GFP (Wang et al., 2001), FER-GFP (Escobar-Restrepo et al., 2007), PIP2A-GFP (Cutler et al., 2000), or GFP-EXO70A1 (Fendrych et al., 2010) were treated in liquid ½ MS medium containing 0.5% DMSO (mock) or 40 μM ES2 for 2 h. Live imaging was performed with a Zeiss LSM 710 Axio Imager laser-scanning confocal microscope operated with Zeiss ZEN software (Carl Zeiss Microscopy LLC, White Plains, NY, USA). The PIN2-GFP, BRI1-GFP, FER-GFP and PIP2A-GFP lines were imaged with a Zeiss Plan-Apochromat 100x/NA1.40 DIC Oil immersion objective at 2048×2048 pixel scanning resolution. The GFP-EXO70A1 line was imaged with a Zeiss Plan-Apochromat 63x/NA1.40 Oil immersion objective at 4096×4096 pixel scanning resolution. The GFP-EXO70A1 seedlings were stained with 10 μg/mL Propidium Iodide (PI) (Biotium Inc., Fremont, CA, USA; Cat # 40016) in ½ liquid MS medium containing DMSO or ES2 in the dark for 10 min, and quickly rinsed in PI-free medium before imaging. GFP was excited with a 488-nm laser line, and PI was excited with a 543-nm laser line. Images were collected from root epidermal cells at the transition zone.

Quantitative image analysis of GFP fluorescence intensity was performed using the FIJI distribution of ImageJ (Ver 1.54b). The regions of interest (ROI) were selected for plasma membrane, cytoplasmic background in the compartment-free region, and the entire cytoplasmic region. The mean fluorescence intensity of the GFP signal for each ROI was measured. The average PM fluorescence intensity was normalized by subtracting the mean fluorescence intensity of the cytoplasmic background. The plasma membrane / cytoplasm ratio was calculated by first subtracting the mean fluorescence intensity of the region outside of the root and then dividing the PM mean fluorescence intensity by the mean fluorescence intensity of the entire cytoplasmic region. Values for individual cells from each seedling were averaged and considered as one data point. Normalization and averaging were performed in Microsoft Excel. Statistical analyses using a Student’s *t*-test were conducted, and box plots were generated with GraphPad Prism software.

## Results

### Plasma membrane enrichment

**Figure 1:**
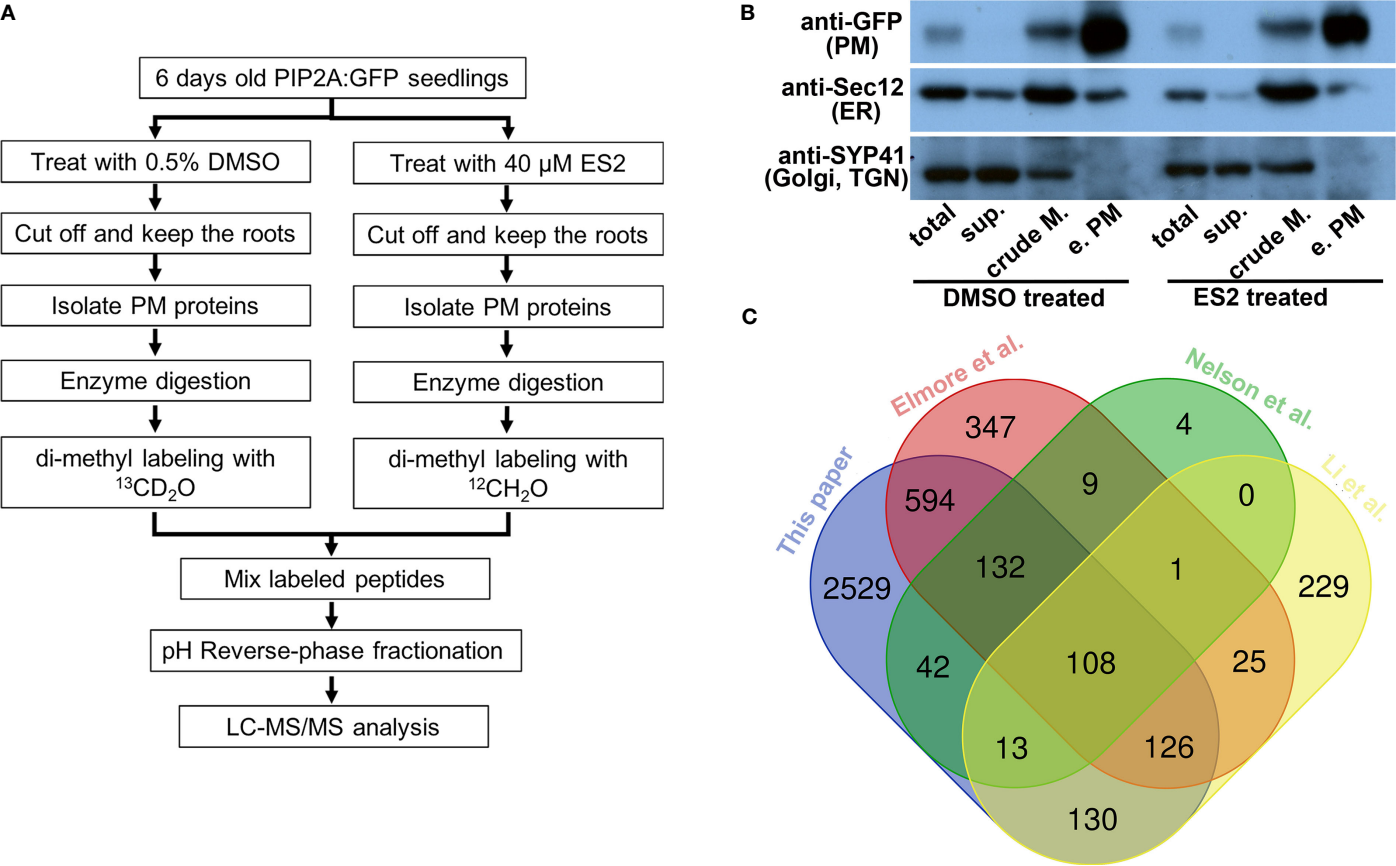
Characterization of enriched plasma membrane fractions shows the high quality of the sample preparation. **(A)** Flowchart of sample preparation for mass spectrometry analysis. **(B)** Western blot characterization of enriched plasma membrane fractions. Protein amount of 12.5 μg from total protein (total), soluble protein (sup.), crude membrane fraction (crude M.) and enriched plasma membrane fraction (e.PM) was separated in SDS-PAGE and transferred to PVDF membrane. The same membrane was blotted with anti-GFP, anti-Sec12 and anti-SYP41 antibodies. **(C)** The Venn diagram comparing the proteins that were identified from different proteomics experiments.

We chose to use a transgenic line expressing PIP2A-GFP as the plant material for our experiments because PIP2A-GFP is localized predominantly at the plasma membrane by microscopy and its cellular localization is not affected by 2 h of 40 μM ES2 treatment (Cutler et al., 2000; Zhang et al., 2016). The GFP tag also allowed the use of anti-GFP antibody to examine the quality of enriched plasma membrane fractions. Six-day-old PIP2A-GFP seedlings were treated with either 40 μM ES2 or 0.5% DMSO for 2 h, a condition in which plant exocytosis is reduced but not completely inhibited (Zhang et al., 2016). After treatment, roots were excised and used for plasma membrane enrichment (**Figure 1A**). We adapted a protocol that allows quick enrichment of plasma membrane and does not require large amounts of plant tissue (Zhang and Peck, 2011; Collins et al., 2017).

Enrichment was evaluated by detecting the presence of proteins known to localize to the plasma membrane (PIP2A-GFP) (Cutler et al., 2000), endoplasmic reticulum (ER) (Sec12) (Bar-Peled and Raikhel, 1997), and Golgi/trans-Golgi network (TGN) (SYP41) (Bassham et al., 2000) in different fractions using Western blots with anti-GFP, anti-Sec12, and anti-SYP41 antibodies, respectively (**Figure 1B**). PIP2A-GFP was enriched in the plasma membrane fraction when compared to the crude membrane fraction (**Figure 1B**, anti-GFP). The ER membrane protein, Sec12, was detected in both crude membrane and plasma membrane fractions; however, the abundance of Sec12 in the plasma membrane fraction was markedly lower than the crude membrane fraction (**Figure 1B**, anti-Sec12). The Golgi/TGN protein, SYP41, was detected in the crude membrane fraction but not in the enriched plasma membrane fraction (**Figure 1B**, anti-SYP41). Immunoblots demonstrated that although proteins from other organelles were present in the enriched plasma membrane fraction, their abundance was substantially reduced in comparison with plasma membrane proteins. We prepared four independent biological replicates of enriched plasma membrane fractions for the mass spectrometry proteomics analysis.

### Mass spectrometry analyses of enriched plasma membrane proteins

From each enriched plasma membrane fraction, 25 μg of protein was digested with Lys-C and trypsin. Resulting peptides from ES2-treated plant samples were dimethyl labeled with ^12^CH_2_O (light, L) and peptides from DMSO-treated samples were dimethyl labeled with ^13^CD_2_O (heavy, H). Dimethyl-labeled peptides from ES2-treatment and control samples were combined and fractionated before LC-MS/MS analysis. The peptides that were detected by LC-MS/MS were assigned to proteins and the abundance of each protein was calculated through a MaxQuant database search. The ratio of each protein’s abundance in differentially labeled samples was calculated using the same process. The data were processed further with the Perseus platform by filtering out rows with invalid values (potential contaminants, the reverse peptides, and peptides only identified by site) from the MaxQuant database search. We further filtered the data by removing proteins with less than three detected values in the four replicates. To compare the data from different replicates, the ratio of protein abundance between differentially labeled proteins was transformed to log_2_ phase and further normalized by median subtraction and then analyzed by histogram (**Supplemental Figure S1**). The normal distribution of log_2_ phase of ratios centered by zero between differentially labeled proteins indicates that the majority of proteins that we detected were not altered by ES2 treatment.

We detected 3675 proteins from four independent replicate experiments (**Supplementary Table S1**). To further evaluate the plasma membrane preparation, we compared our protein list with those published from different Arabidopsis tissues using another plasma membrane preparation method (**Figure 1C**). First, we compared our plasma membrane protein list with one generated from two-week-old liquid cultured *Arabidopsis* seedlings using a two-phase partition of microsomes and Trypsin-catalyzed ^18^O labeling (Nelson et al., 2006). We found 295 overlapping proteins, accounting for 95% of the proteins detected in Nelson et al.’s experiments (309 proteins in total) (**Figure 1C**). Elmore et al. used an aqueous two-phase partitioning approach for leaves of four-week-old Arabidopsis plants grown in soil condition and consistently identified more than 1300 proteins in replicate experiments (Elmore et al., 2012). We found 960 overlapping proteins, accounting for more than 70% of the proteins detected in Elmore et al.’s experiments (**Figure 1C**). Li et al. isolated plasma membrane fractions from Arabidopsis suspension-cultured cells using a two-phase partition method and identified over 600 proteins by proteomic analysis (Li et al., 2012). About 60% of the proteins that were detected from cultured cells were consistently identified in our experiments (**Figure 1C**). Substantial overlap between our plasma membrane protein list and others that use a different method or tissue indicates that our proteomic data can be used to compare the effects of ES2 on the abundance of plasma membrane proteins.

To examine whether the enriched plasma membrane fraction was free from contaminants from other large membrane-bound compartments, we checked our proteomics list for the presence of proteins residing in the nuclear envelope and vacuole. The nuclear envelope protein SAD1/UNC-84 DOMAIN PROTEIN 1 (SUN1, AT5G04990; Oda and Fukuda, 2011) was detected, but its abundance was unchanged following ES2 treatment, with a mean log2 fold-change of 0.00. Another nuclear envelope protein SAD1/UNC-84 DOMAIN PROTEIN 2 (SUN2, AT3G10730; Oda and Fukuda, 2011) was not detected. The vacuole membrane protein TONOPLAST INTRINSIC PROTEIN 1;1 (γ-TIP or TIP1;1, AT2G36830; Nelson et al., 2006) was not detected. These results indicate that the enriched plasma membrane fraction was relatively free from contamination of nuclear envelope or vacuolar membranes.

To investigate whether ES2 treatment altered the membrane identity of the plasma membrane fraction, we checked our proteomics list for the presence of several markers of membrane-bound organelles. The autophagosome markers ATG8e (AT2G45170) and ATG8f (AT4G16520) were not detected. The early endosome marker ARA6 (AT3G54840; Ebine et al., 2011), ER marker SEC12 (AT2G04170; Bar-Peled and Raikhel, 1997), and Golgi marker SYP32 (AT3G24350) and ALPHA-MANNOSIDASE 1 (ManI, AT1G51590; Kajiura et al., 2010) were all present but unchanged following ES2 treatment, with a mean log2 fold-change of 0.06 for ARA6, 0.05 for SEC12, 0.00 for SYP32, and -0.01 for ManI (**Supplementary Table S1**). These results indicate that the membrane identify of the plasma membrane was not altered following ES2 treatment.

To identify proteins with altered abundance after ES2 treatment, we performed a moderated t-test (Smyth, 2004) for each protein using the limma package in R. The moderated t-test uses a hierarchical empirical Bayesian model to shrink the sample standard deviation estimates for each protein towards a pooled value. This stabilizes the t-statistics and increases the power of the test to detect true differences in protein abundance. Since multiple significance tests were performed, we used the Benjamini-Hochberg procedure to bound the expected false-discovery rate at 10%. After the correction, 330 proteins had adjusted p-values less than 0.10. Thus, we can estimate that roughly 300 of the 330 proteins have log_2_ ratios significantly different from 0. Among these 330 proteins, 145 had reduced abundance (**Supplementary Tables S2**, **S3**) and 185 had increased abundance (**Supplementary Table S4**) in the plasma membrane fraction of ES2-treated samples. To highlight the proteins with the most dramatic changes, the top twenty proteins with decreased and increased abundance were listed in **Tables 1**, **2**, respectively. Because ES2 binds to the EXO70A1 subunit of the exocyst complex and inhibits its function (Zhang et al., 2016), and because EXO70A1 serves as a landmark for exocyst complex recruitment to the plasma membrane (Synek et al., 2021), we expect that proteins with reduced abundance at the plasma membrane are candidate cargos of exocyst-mediated trafficking.

**Table 1:** Top 20 proteins with reduced abundance in enriched plasma membrane fractions after a 2-h ES2 treatment and their Gene Ontology terms in biological processes category.

Protein ID	Protein Name	Protein Symbol	Mean L/H	Representative GO Terms (Biological Processes)	No. of TM Helices	Presence of signal peptide
AT1G66230	MYB DOMAIN PROTEIN 20	ATMYB20	0.02	regulation of secondary cell wall biogenesis (GO:2000652)	0	NO
AT3G16630	Kinesin-like protein KIN-13A	KIN13A	0.09	microtubule depolymerization (GO:0007019)	0	NO
AT3G61480	Quinoprotein amine dehydrogenase, beta chain-like RIC1-like guanyl-nucleotide exchange factor		0.10	intracellular protein transport (GO:0006886)	0	NO
AT3G22240	CYSTEINE-RICH TRANSMEMBRANE MODULE 9	ATHCYSTM9	0.26	indole-containing compound biosynthetic process (GO:0042435)	0	NO
AT3G08610	NADH dehydrogenase ubiquinone 1 alpha subcomplex subunit		0.30	catabolic process (GO:0009056)	1	NO
AT4G36670	POLYOL/MONOSACCHARIDE TRANSPORTER 6	ATPMT6	0.31	response to temperature stimulus (GO:0009266)	12	NO
AT3G45650	Protein NRT1/PTR FAMILY 2.7	NPF2.7	0.35	GO:0015698	12	NO
AT5G25820	Exostosin family protein		0.35	root morphogenesis (GO:0010015)	1	NO
AT1G65730	Probable metal-nicotianamine transporter YSL7	YSL7	0.37	catabolic process (GO:0009056)	17	NO
AT1G12950	Protein DETOXIFICATION 31	DTX31	0.38	root hair elongation (GO:0048767)	12	NO
AT2G25980	JACALIN-RELATED LECTIN 20	JAL20	0.38		0	NO
AT5G35940	Mannose-binding lectin superfamily protein		0.38	organic acid metabolic process (GO:0006082)	0	NO
AT5G40780	LYSINE HISTIDINE TRANSPORTER 1	LHT1	0.39	amino acid transmembrane transport (GO:0003333)	11	NO
AT1G17260	AUTOINHIBITED H(+)-ATPASE 10	AHA10	0.40	proton transmembrane transport (GO:1902600)	10	NO
AT2G01520	MLP-LIKE PROTEIN 328	MLP328	0.40	defense response (GO:0006952)	0	NO
AT2G39510	USUALLY MULTIPLE ACIDS MOVE IN AND OUT TRANSPORTERS 14	UMAMIT14	0.40	L-glutamate import across plasma membrane (GO:0098712)	10	NO
AT5G26260	TRAF-like family protein		0.40	plant epidermis development (GO:0090558)	0	YES
AT3G20380	TRAF-like family protein		0.41		0	YES
AT1G14160	CASP-LIKE PROTEIN 1A1	CASPL1A1	0.42	cell wall modification (GO:0042545)	4	NO
AT1G50630	Extracellular ligand-gated ion channel protein (DUF3537)		0.42	root development (GO:0048364)	7	NO

The L/H value is the ratio of the abundance in ES2-treated samples divided by the abundance in mock-treated samples. The number of TM helices and presence of signal peptide were predicted by the DeepTMHMM package.

**Table 2:** Top 20 proteins with increased abundance in enriched plasma membrane fractions after a 2-h ES2 treatment and their Gene Ontology terms in biological processes category.

Protein ID	Protein Name	Protein Symbol	Mean L/H	Representative GO Term (Biological Processes)	No. of TM Helices	Presence of signal peptide
AT4G14320	Zinc-binding ribosomal protein family protein	RPL36AB	13.84	translation (GO:0006412)	0	NO
AT1G15520	ATP-BINDING CASSETTE G40	ABCG40/ PDR12	10.93	abscisic acid transport (GO:0080168)	12	NO
AT1G80660	H(+)-ATPASE 9	AHA9	10.25	proton transmembrane transport (GO:1902600)	10	NO
AT3G56860	UBP1-ASSOCIATED PROTEIN 2A	UBA2A	8.33	abscisic acid-activated signaling pathway (GO:0009738)	0	NO
AT1G33680	KH domain-containing protein	ATKH3	7.77	regulation of gene expression (GO:0010468)	0	NO
AT5G42380	CALMODULIN LIKE 37	CML37	7.35	response to water deprivation (GO:0009414)	0	NO
AT1G20880	RNA-binding (RRM/RBD/RNP motifs) family protein		7.10	biological_process_unknown (GO:0008150)	0	NO
AT1G78260	RNA-binding (RRM/RBD/RNP motifs) family protein		7.07	plant epidermis development (GO:0090558)	0	NO
AT5G18150	Methyltransferase-related protein		7.00	regulation of defense response (GO:0031347)	0	NO
AT3G13060	EVOLUTIONARILY CONSERVED C-TERMINAL REGION 5	ECT5	6.96	mRNA destabilization (GO:0061157)	0	NO
AT4G03110	RNA-BINDING PROTEIN-DEFENSE RELATED 1	RBP-DR1	6.78	plant-type hypersensitive response (GO:0009626)	0	NO
AT3G07660	flocculation protein (DUF1296)		6.37	biological_process_unknown (GO:0008150)	0	NO
AT5G56670	Ribosomal protein S30 family protein	RPS30C	5.57	translation (GO:0006412)	0	NO
AT4G36960	RNA-binding (RRM/RBD/RNP motifs) family protein		5.46	organic cyclic compound catabolic process (GO:1901361)	0	NO
AT3G13222	GBF-INTERACTING PROTEIN 1	GIP1	5.31	positive regulation of DNA binding (GO:0043388)	0	NO
AT3G13460	EVOLUTIONARILY CONSERVED C-TERMINAL REGION 2	ECT2	5.27	mRNA destabilization (GO:0061157)	0	NO
AT1G29350	RNA polymerase II degradation factor-like protein (DUF1296)		5.11	regulation of nitrogen compound metabolic process (GO:0051171)	0	NO
AT2G38540	LIPID TRANSFER PROTEIN 1	ATLTP1	5.09	lipid transport (GO:0006869)	0	YES
AT4G22670	HSP70-INTERACTING PROTEIN 1	HIP1	4.98	chaperone cofactor-dependent protein refolding (GO:0051085)	0	NO
AT2G46780	RNA-binding (RRM/RBD/RNP motifs) family protein		4.93	plant epidermis development (GO:0090558)	0	NO

The L/H value is the ratio of the abundance in ES2-treated samples divided by the abundance in mock-treated samples. The number of TM helices and presence of signal peptide were predicted by the DeepTMHMM package.

### Confocal imaging of GFP-tagged plasma membrane proteins following ES2 treatment

**Figure 2:**
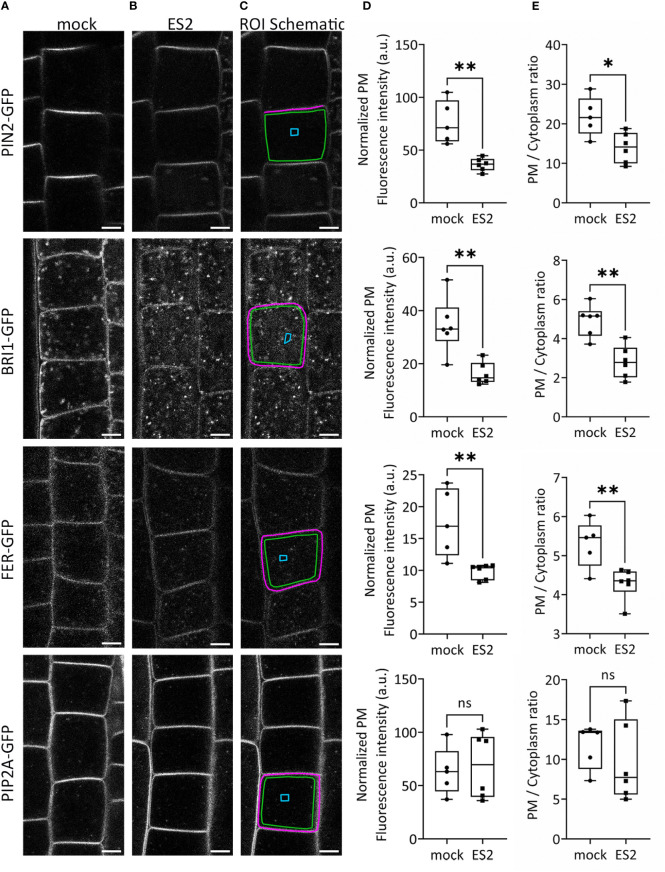
The abundance of several proteins at the plasma membrane is reduced following ES2 treatment. **(A, B)** Representative confocal microscope images of root epidermal cells from 6-d-old transgenic seedlings expressing PIN2-GFP, BRI1-GFP, FER-GFP or PIP2A-GFP following a 2-h mock treatment **(A)** or a 40 μM ES2 treatment **(B)**. **(C)** Schematic illustration of the region of interest (ROI) selection. The plasma membrane (PM) ROI is indicated by a magenta line; cytoplasmic background in the compartment-free subregion is indicated by a blue box (used as the background for normalization); the entire cytoplasmic region is indicated by a green box. Scale bar = 5 μm for all images. **(D)** Quantification of the normalized average PM fluorescence intensity from images similar to those shown in **(A**, **B)** The average PM fluorescence intensity was normalized by subtracting the mean fluorescence intensity of the cytoplasmic background. **(E)** Quantification of the average PM/cytoplasm fluorescence intensity ratio. The PM/cytoplasm mean fluorescence intensity ratio was calculated by first subtracting the mean fluorescence intensity of the region outside of the root as the imaging background, and then dividing the PM mean fluorescence intensity by the entire cytoplasm mean fluorescence intensity. (N = 21–75 cells from 5–7 seedlings). Statistical analysis was performed with an unpaired two-tailed Student’s *t*-test, * denotes P value < 0.05, ** denotes P value < 0.01, ns denotes not significant. In the box plot, whiskers indicate the maximum and minimum values of the dataset, whereas individual data points represent the average value from each seedling.

To confirm the identification of proteins with reduced abundance after ES2 treatment, we performed live imaging of GFP-tagged plasma membrane proteins in Arabidopsis root epidermal cells using laser scanning confocal microscopy (**Figure 2**). We selected three protein candidates: the auxin efflux carrier protein PIN-FORMED 2 (PIN2), the leucine-rich-repeat receptor kinase of brassinosteroid signaling BRASSINOSTEROID INSENSITIVE 1 (BRI1), and the receptor kinase FERONIA (FER), each of which had reduced plasma membrane abundance after ES2 treatment in our proteomics analysis. PIN2 had a mean log2 fold-change of -0.27, BRI1 had a mean log2 fold-change of -0.31, and FER had a mean log2 fold-change of -0.38. (**Supplementary Table S1**). We quantified GFP-tagged protein fluorescence intensity on the plasma membrane and normalized by subtracting the mean background fluorescence intensity from a cytoplasmic compartment-free region (**Figure 2C**). The abundance of all three proteins at the plasma membrane region were significantly decreased after a 2-h ES2 treatment (**Figure 2D**). To further evaluate the change of abundance between the plasma membrane and entire cytoplasmic region, we also quantified the plasma membrane / cytoplasm intensity ratio as another indicator of plasma membrane protein abundance (**Figure 2E**). The plasma membrane / cytoplasm intensity ratios for all three proteins were significantly reduced after a 2-h ES2 treatment compared to mock treatment (**Figure 2E**). Our analysis showed for the first time that the plasma membrane abundance of FER was decreased following ES2 treatment. As a negative control, we measured the abundance of PLASMA MEMBRANE INTRINSIC PROTEIN 2A (PIP2A), which was shown to have unaltered plasma membrane abundance after ES2 treatment in the proteomics analyses, with a mean log2 fold-change of 0.05 (**Supplementary Table S1**). The plasma membrane abundance and plasma membrane / cytoplasm intensity ratio of PIP2A-GFP were not altered by ES2 treatment (**Figures 2D, E**).

### Gene Ontology enrichment analysis of candidate cargos for ES2-targeted trafficking

**Figure 3:**
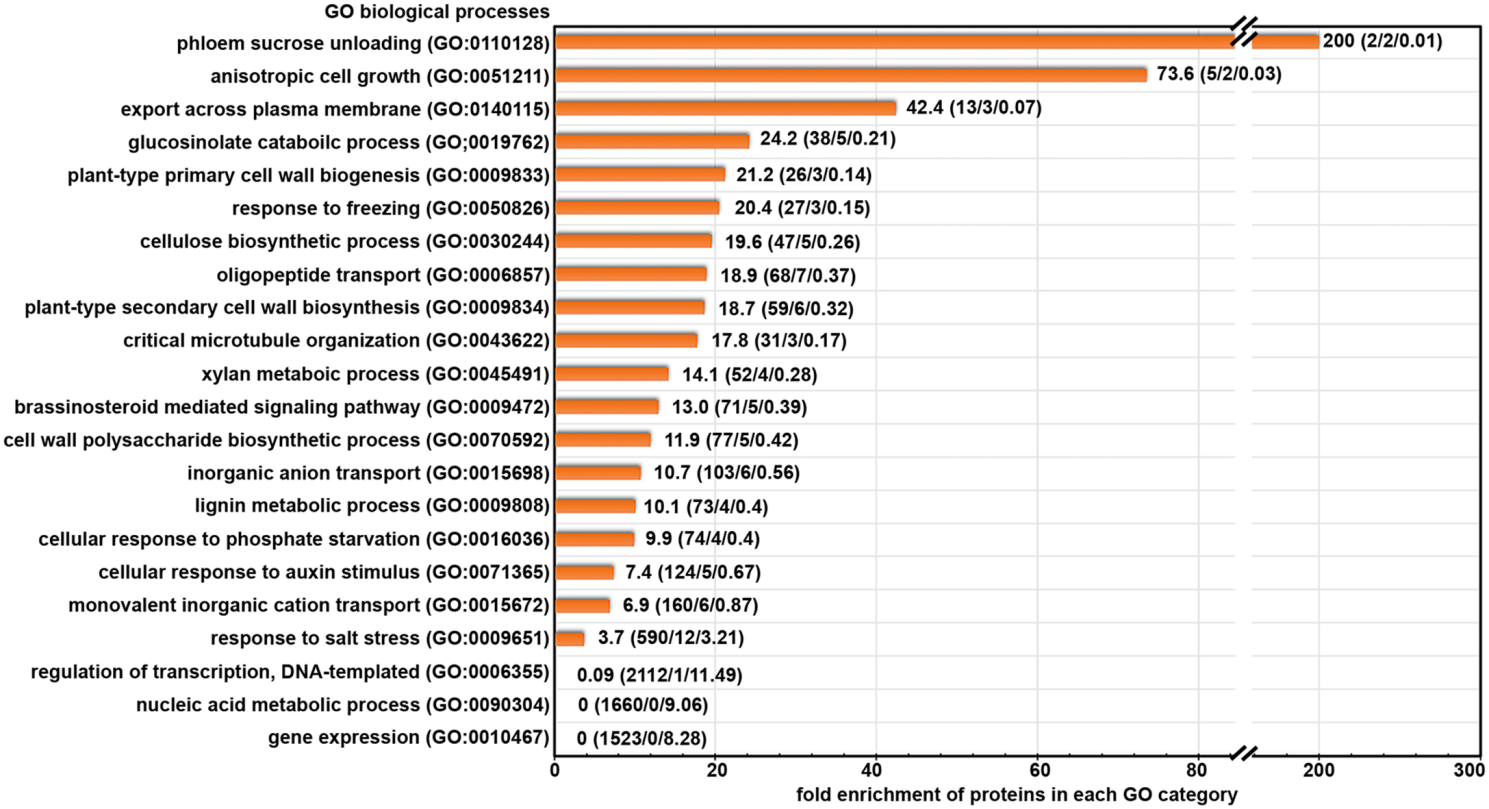
Gene Ontology terms of biological processes category that are enriched in our protein list with significantly reduced abundance after ES2 treatment. Numbers on each row represent: fold enrichment (total number of proteins in the category/number of proteins identified in the reduced protein list/number of proteins expected in reduced protein list). Fold enrichment represents the ratio of the number of proteins identified divided by the number of proteins expected in the list.

To better understand the biological functions of the candidate cargo proteins for ES2-targeted trafficking, we performed Gene Ontology (GO) enrichment analysis of the biological processes category on the 145 proteins with reduced abundance at the plasma membrane using the PANTHER classification system (Mi et al., 2013; Mi et al., 2017). We found that 49 proteins could be classified into protein families that were enriched in abundance on our list when compared to their abundance in the reference list of *Arabidopsis thaliana* genome (**Figure 3**). The fold enrichment for different biological processes ranged from 3.7 to 200. The 49 candidate exocytosis cargo proteins belong to gene families that function in plant cell growth, cell wall biosynthesis, hormone signaling, response to stresses and membrane transport (**Figure 3**; **Supplementary Table S2**). The proteins for the regulation of DNA-templated transcription (GO:0006355), nucleic acid metabolic process (GO:0090304) and gene expression (GO:0010467) were much lower than the numbers that were expected from the Arabidopsis genome (**Figure 3**), indicating the trafficking of these proteins was not mediated by the exocyst complex.

We further examined the annotated functions of proteins with significantly reduced abundance in ES2-treated samples. We found the abundance of three primary cell wall cellulose synthases (CESA1, CESA3, and CESA6), CELLULOSE SYNTHASE-INTERACTIVE PROTEIN 1 (CSI1), and KORRIGAN (KOR) was significantly reduced in ES2-treated samples (**Supplementary Table S2**). Proteins involved in lignin metabolism were also reduced, as were multiple proteins involved in brassinosteroid and auxin signaling. The abundance of some proteins that respond to cold and phosphate deficiency was significantly reduced in ES2-treated samples. Another group of proteins that was affected by ES2 treatment are transporters responsible for the movement of phosphate, sulphate, potassium, and oligopeptides across the plasma membrane. The abundance of two annexins involved in phloem sucrose unloading (Wang et al., 2018) was also significantly reduced (**Supplementary Table S2**).

In addition to the proteins mentioned above, there are other proteins with reduced abundance at the plasma membrane, but they were not enriched in functional groups in the GO enrichment analysis. These include multiple receptor-like protein kinases, transporters, enzymes for carbohydrate metabolism and protein glycosylation, as well as proteins with uncharacterized functions. Although these proteins were not enriched in our list, they may have diverse functions and cellular localization patterns among different members of the family. Collectively, our GO term enrichment analysis indicates that the exocyst complex mediates constitutive transport of numerous proteins that function in cell growth, cell wall biosynthesis, hormone signaling, stress response, and membrane transport.

### Bioinformatic analysis of potential cargo proteins for plant exocyst complex

Plants use both conventional and unconventional trafficking pathways to deliver proteins to the plasma membrane and extracellular space (Drakakaki and Dandekar, 2013; Ding et al., 2014; Robinson et al., 2016; Aniento et al., 2022). In the conserved conventional trafficking pathway, proteins with a signal peptide are inserted into the endoplasmic reticulum during translation and are then transported to Golgi through vesicles and further secreted through post-Golgi vesicles (Novick et al., 1981; Rothman, 2002; Schekman, 2010). However, increasing evidence from proteomic analysis shows that many proteins that are secreted out of the cell or targeted to the plasma membrane do not have signal peptides, indicating the existence of unconventional trafficking pathways for these proteins (Oh et al., 2005; Soares et al., 2007; Wen et al., 2007; Han et al., 2010; Chen et al., 2016). Plant exocyst complex has been shown to regulate cargo proteins through both conventional and unconventional pathways (Wang et al., 2010; Drdová et al., 2013; Lin et al., 2015; Mayers et al., 2017).

To test whether the candidate cargo proteins contain signal peptides that allow them to traffic through the conventional pathway, we analyzed the presence of signal peptides in proteins that had reduced abundance in ES2-treated samples using the DeepTMHMM package, a deep-learning method for signal peptide and transmembrane domain prediction (Hallgren et al., 2022). DeepTMHMM predicted that 36 of the 145 proteins with reduced abundance contained signal peptides (**Supplementary Table S5**). The existence of a signal peptide in a small percentage of proteins indicates that consistent with previous findings, plant exocyst complex mediates the transport of proteins in both conventional and unconventional trafficking pathways. To understand how these candidate cargo proteins are associated to the plasma membrane, we analyzed the presence of potential transmembrane domains in these 145 proteins using the DeepTMHMM package. We found 64 of the 145 proteins were predicted to have at least one transmembrane domain (**Supplementary Table S5**).

### The behavior of the exocyst complex and its regulators is different than candidate cargo proteins

All eight subunits of the exocyst complex were detected in the enriched plasma membrane fractions (**Supplementary Table S1**). However, we did not find a statistically significant reduction in their abundance in ES2-treated samples (**Supplementary Table S1**). Among the eight exocyst subunits, only SEC3 showed a 13% reduction in the mean value after ES2 treatment but was not statistically different (**Supplementary Table S1**). STOMATAL CYTOKINESIS-DEFECTIVE 1 (SCD1), a component of the candidate activator of plant RabE GTPase that regulates the exocyst complex (Mayers et al., 2017), was detected in plasma membrane fractions but its abundance was not altered by ES2 treatment (**Supplementary Table S1**). We identified 12 Syntaxins of Plants (SYP), SYP111, SYP121, SYP122, SYP132, SYP21, SYP22, SYP32, SYP43, SYP51, SYP52, SYP61, and SYP71, but none of these were statistically significantly reduced (**Supplementary Table S1**). In summary, our proteomics results indicate that the abundance of candidate tSNARE proteins, together with the exocyst complex and the SCD complex, were not significantly affected by a 2-h, 40 μM ES2 treatment.

### Confocal imaging of GFP-EXO70A1 following ES2 treatment

**Figure 4:**
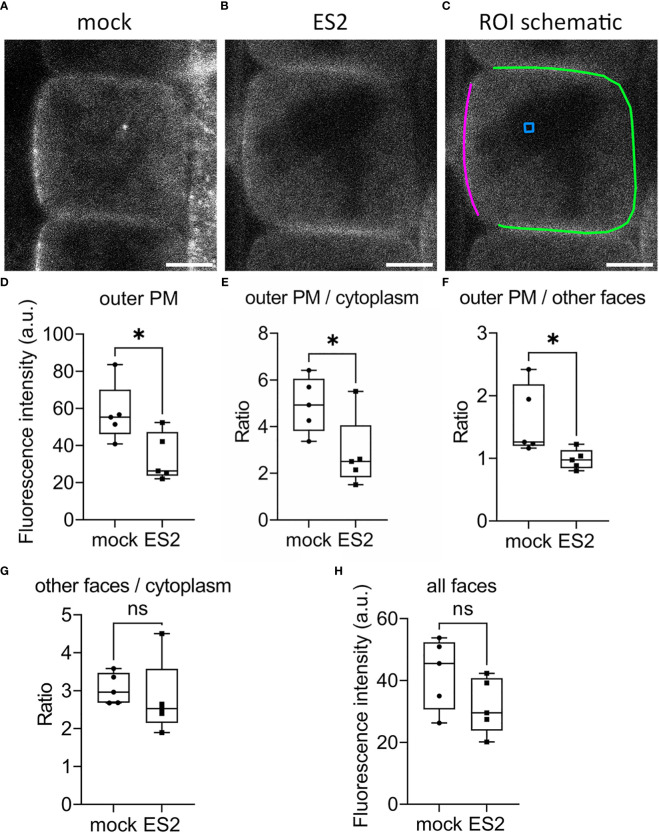
The abundance of EXO70A1 at the plasma membrane on the outer periclinal face is decreased following ES2 treatment. **(A, B)** Representative confocal microscopy images of root epidermal cells from 6-d-old transgenic seedlings expressing GFP-EXO70A1 following a 2-h mock treatment **(A)** or a 40 μM ES2 treatment **(B)**. Scale bar = 5 μm for all images. **(C)** Schematic illustration of the region of interest (ROI) selection. The plasma membrane on the outer periclinal face was marked with a magenta line, whereas the plasma membrane of the other three faces was marked with a green line. The cytoplasmic background in the vacuole or nucleus region (used for background subtraction) was indicated with a blue box. **(D)** Quantification of the average GFP-EXO70A1 fluorescence intensity at the plasma membrane on the outer periclinal face (as marked with a magenta line in **C**) from images similar to those shown in **(A**, **B, E)** Quantification of the ratio of fluorescence intensity of GFP-EXO70A1 at the plasma membrane on the outer periclinal face compared to the entire cytoplasmic region after normalization. **(F)** Quantification of the ratio of GFP-EXO70A1 fluorescence intensity at the plasma membrane on the outer periclinal face compared to the other three faces (marked with a green line in **C**). **(G)** Quantification of the ratio of fluorescence intensity of GFP-EXO70A1 at the plasma membrane on the other three faces compared to the entire cytoplasmic region after normalization. **(H)** Quantification of the average fluorescence intensity of GFP-EXO70A1 at the plasma membrane on all visible faces of the plasma membrane in medial views of the epidermal cell. Fluorescence intensities at the plasma membrane and entire cytoplasmic region were normalized by subtracting the cytoplasmic background as indicated with a blue box in **(C)** (N = 17–23 cells from 5 seedlings). Statistical analysis was performed with an unpaired two-tailed Student’s *t*-test, * denotes P value < 0.05, ns denotes not significant. In the box plot, whiskers indicate the maximum and minimum values of the dataset, whereas individual data points represent the average value from each seedling.

To investigate whether ES2 affects the spatial distribution or abundance at the plasma membrane of its target, EXO70A1, we performed live imaging of root epidermal cells from GFP-EXO70A1 seedlings using laser scanning confocal microscopy (**Figure 4**). Consistent with previous findings (Zhang et al., 2016; Larson et al., 2020; Synek et al., 2021), EXO70A1 in mock-treated epidermal cells showed a polarized distribution with highest abundance at the plasma membrane on the outer periclinal face, but was present at all six faces of the cell (**Figure 4A**). A 2-h, 40 µM ES2 treatment significantly reduced the abundance of EXO70A1 at the plasma membrane on the outer periclinal face (**Figures 4B, D**), and disrupted the polarized distribution of EXO70A1 at the outer periclinal face when fluorescence intensity was expressed as a ratio with the cytosolic region (**Figure 4E**) or as a ratio with the other faces of the same cell (**Figure 4F**). However, when EXO70A1 at the other three faces of the plasma membrane visible in medial optical sections, including the inner periclinal face and the two anticlinal faces (green line in **Figure 4C**), was examined, the apparent abundance expressed as a ratio with cytosol was not affected by ES2 treatment (**Figure 4G**). When all four faces of the plasma membrane were combined and averaged, there was a modest decrease in the abundance of EXO70A1, but this was not statistically significant (**Figure 4H**). In total, a 2-h ES2 treatment had greatest effect on the polarized distribution of EXO70A1 and its association with the plasma membrane at the outer periclinal face of root epidermal cells, but a rather modest reduction in overall abundance at the plasma membrane on all faces of the cell.

### Gene Ontology enrichment analysis of proteins with increased abundance after ES2 treatment

Our statistical analysis identified 185 proteins with increased abundance in enriched plasma membrane fractions of ES2-treated samples (**Supplementary Table S4**). To better understand the function of these proteins, we performed Gene Ontology enrichment analysis of the biological function category. We found that this list of proteins is enriched in biological functions of COPII-coated vesicle cargo loading, mRNA catabolic process, negative regulation of translation, proton membrane transport, aerobic respiration, leaf senescence, ribonucleoprotein complex biogenesis, and amide biosynthesis process (**Supplementary Figure S3**). The functional relationship between exocyst complex and these proteins has not been well characterized. We speculate that some of these proteins might be involved in cellular responses to the inhibition of exocytosis.

## Discussion

### Exocyst complex mediates constitutive transport of proteins with diverse functions during plant growth and development

Using quantitative and comparative proteomics analysis, we identified 145 proteins with significantly reduced abundance at the plasma membrane of Arabidopsis roots following ES2 treatment. Some of these proteins possess transmembrane domains and are likely integral membrane proteins delivered to the plasma membrane by secretion. The candidate proteins without a predicted transmembrane domain could be associated with the plasma membrane through lipid anchors, through interaction with other membrane proteins, or by association with polar head groups of the lipid bilayer. These candidates of exocyst-mediated trafficking perform functions in cell growth, cell wall biosynthesis, hormone signaling, stress responses, membrane transport, nutrient transport, and protein phosphorylation. The dynamics of many hormone-signaling proteins are well characterized; however, the role of exocyst complex in regulating their trafficking awaits further study. The functional diversity of proteins that are transported through the exocyst complex is consistent with the pleiotropic phenotypes observed in plants deficient for the exocyst complex (Cole et al., 2005; Synek et al., 2006; Hála et al., 2008; Fendrych et al., 2010; Kulich et al., 2010; Wang et al., 2010; Pečenková et al., 2011; Drdová et al., 2013; Zhang et al., 2013; Kulich et al., 2015; Drdová et al., 2019; Ogura et al., 2019; Synek et al., 2021; Hématy et al., 2022). Our results indicate that the plant exocyst complex plays essential roles for growth and adaptation by mediating constitutive transport of proteins with diverse functions to the plasma membrane.

Some other important plasma membrane proteins, such as Rho family small GTPases (ROPs), multidrug resistance-like ABC transporter family proteins, and aquaporin plasma membrane intrinsic proteins were not affected by ES2 treatment in this proteomics study (**Supplementary Table S1**), which is consistent with the previous live-cell imaging results (Zhang et al., 2016). Our current results indicate that these important plasma membrane proteins could have slow turnover rates or are not delivered to the plasma membrane through the ES2-targeted exocyst complex.

### Cargo protein exocytosis and exocyst complex membrane association

Time-lapse imaging revealed that the plant exocyst complex colocalizes with its cargo protein (Cellulose Synthase Complex or CSC) during the tethering process and then separates from the cargo after successful fusion (Zhu et al., 2018; Zhang and Staiger, 2021; Zhang et al., 2021). In our proteomics study, we detected a significant reduction in the abundance of cargo proteins, such as cellulose synthases, but did not detect a statistically significant reduction in the abundance of the exocyst complex subunits. The abundance of SCD complex components and candidate tSNAREs on the plasma membrane was also not affected by ES2 treatment. Previous live-cell imaging data reveal that exocyst subunits are polarized to the outer periclinal face of root epidermal cells (Zhang et al., 2016; Larson et al., 2020; Synek et al., 2021) and are disrupted by ES2 treatment (Zhang et al., 2016) or genetic disruption of EXO70A1 (Synek et al., 2021), but not by genetic disruption of some SNARE proteins that physically interact with the exocyst complex (Larson et al., 2020). The plasma membrane enrichment reflects the overall abundance of each plasma membrane protein, but not their polarized distribution on a specific face of the plasma membrane. Results from live-cell imaging and our proteomics analyses indicated that ES2 treatment affected the polarized distribution of the exocyst complex in root epidermal cells but did not markedly affect its association with the plasma membrane. The lifetime of exocyst foci on the plasma membrane of root epidermal cells is around 10 s; however, inhibition of exocytosis by a 2-h Brefeldin A (BFA) treatment did not affect membrane localization of the exocyst complex (Fendrych et al., 2013). Thus, exocyst complex association with the plasma membrane may not depend on the other components of the exocytosis machinery.

Previous studies demonstrate that exocyst subunits physically interact and associate with the SCD1/2 complex (Mayers et al., 2017), and EXO70 physically interacts with several SNAREs (Larson et al., 2020). Although ES2 disrupts the polarized localization of EXO70A1, our current data do not directly reflect whether ES2 disrupts the physical interaction of exocyst subunits with SCD1/2 complex or SNARE proteins, and/or affects the localization pattern of SCD1/2 and SNAREs. One future direction would be to quantify the localization of fluorescent-tagged SCD1/2 and SNAREs following ES2 treatment. Further, it would be interesting to quantify the association of exocyst subunits with other components of the exocytosis machinery through high spatial-temporal resolution live-cell imaging of double marker lines.

### Future directions in understanding plant plasma membrane protein dynamics

Due to the importance of plant plasma membrane proteins, multiple comparative proteomic studies have been conducted to investigate the profiles of plasma membrane proteins in response to environmental stresses and cell growth status. The composition of plasma membrane proteins changes upon salt stress, cold treatment, osmotic stress, immune signaling, nutrient deficiency and different growth stages of cultured cells (Nohzadeh Malakshah et al., 2007; Nouri and Komatsu, 2010; Elmore et al., 2012; Li et al., 2012; Cao et al., 2016; Chen et al., 2016; Li et al., 2018). These proteomic studies show that the plasma membrane serves as a hub for cells to respond to environmental and growth cues. These studies further indicate that the vesicle trafficking machinery that facilitates the transport of these proteins is essential for plants to respond to biotic and abiotic stresses. Previous findings on the essential functions of plant exocyst complex in plant growth and immune responses (Synek et al., 2006; Fendrych et al., 2010; Kulich et al., 2010; Kulich et al., 2015; Cole et al., 2018; Du et al., 2018; Guo et al., 2018; Žárský, 2022) support the hypothesis that the exocyst complex choreographs the dynamic change of specific proteins at the plasma membrane in response to different environmental stimuli and growth cues.

In comparison with yeast and mammalian cells, we understand less about the mechanisms of plasma membrane protein delivery and turnover in plants. In this study, using very stringent filtering and cutoff, we found 145 proteins that have significantly (at 10% FDR) reduced abundance at the plasma membrane after a 2-h, 40 μM ES2 treatment, a condition that inhibits but does not completely arrest exocytosis and plant growth. We found that the abundance of some plant plasma membrane proteins was reduced by more than 50%, indicating that the half-time of turnover of these proteins is less than 2 h. We speculate that these proteins undergo fast delivery to the plasma membrane mediated by the exocyst complex and have a rapid turnover at the plasma membrane. There are other proteins that have lower abundance in ES2-treated samples but did not pass the statistical test. We believe these proteins have slower delivery and/or turnover rates at the plasma membrane. It is not well understood how plants control precise delivery of cargo proteins with different dynamics.

## Conclusion

Using a 2-h treatment with the exocyst subunit EXO70A1 inhibitor ES2 in combination with plasma membrane enrichment, quantitative proteomic analyses, and live-cell imaging, we identified subsets of plasma membrane proteins that require the ES2-targeted exocyst complex for constitutive trafficking to the plasma membrane in Arabidopsis root cells. Our dataset provides new insights for the identification of novel cargo proteins of the exocyst complex.

## Data availability statement

The datasets presented in this study can be found in online repositories. The names of the repository/repositories and accession number(s) can be found below: ProteomeXchange Consortium via the PRIDE database, with the identifier of PXD037455.

## Author contributions

CZ and XL: conceptualization. XL, PZ, Y-JC, LH, and DW: investigation. XL and DN: formal analysis. XL, PZ, C-CH, GL, WAT, and CZ: methodology. CZ and WAT: resources. XL and CZ: data curation, visualization, and validation. CZ, XL, and CJS: writing - original draft. PZ, Y-JC, DN, LH, DW, C-CH, GL, and WAT: writing - review & editing. CZ, GL, WAT, and CJS: supervision. CZ and XL: funding acquisition. CJS: agrees to serve as the author responsible for contact and ensures communication. All authors contributed to the article and approved the submitted version.
