# Supplementary material for: The EXO70 inhibitor Endosidin2 alters plasma membrane protein composition in Arabidopsis roots
Source: Front Plant Sci. 2023 May 31;14:1171957. doi: 10.3389/fpls.2023.1171957 (PMC10264680; doi:10.3389/fpls.2023.1171957)
Supplement: Supplementary file 1 [file DataSheet_1.pdf]

Supplementary tables and figures for the manuscript:

Plant exocyst complex mediates transport of a diverse array of proteins to the plasma membrane

Xiaohui Li[1,2], Peipei Zhu[4,5], Yen-Ju Chen[1,2], Lei Huang[1,2], Diwen Wang[1,2], David T. Newton[6], Chuan-Chih Hsu[4], Guang Lin[7,8], W. Andy Tao[4,5], Christopher J. Staiger[1,2,3*] and Chunhua Zhang[1,2]

[1] Department of Botany and Plant Pathology, 915 W. State Street, Purdue University, West Lafayette, IN, 47907, USA
[2] Center for Plant Biology, 615 W. State St, Purdue University, West Lafayette, IN, 47907, USA
[3] Department of Biological Sciences, 915 W. State Street, Purdue University, West Lafayette, IN, 47907, USA
[4] Department of Biochemistry, Purdue University, 75 S. University St, West Lafayette, IN, 47907, USA
[5] Department of Chemistry, Purdue University, 575 W. Stadium Ave, West Lafayette, IN 47907, USA
[6] Department of Statistics, Purdue University, 150 N. University St, West Lafayette, IN 47907, USA
[7] Department of Mathematics, Purdue University, 150 N. University Street, West Lafayette, IN 47907, USA
[8] School of Mechanical Engineering, Purdue University, 585 Purdue Mall, West Lafayette, IN 47907, USA

[*] **Correspondence:**
Christopher J. Staiger
Email: staiger@purdue.edu

Supplementary Table S1. Summary of proteins identified in mass spectrometry analyses.

Supplementary Table S2. Proteins with 20% or more reduction in abundance in enriched plasma membrane fractions after a 2-h ES2 treatment and their Gene Ontology terms in biological processes category.

Supplementary Table S3. Raw counts of proteins with significantly decreased abundance in ES2-treated samples.

Supplementary Table S4. Raw counts of proteins with significantly increased abundance in ES2-treated samples.

Supplementary Table S5. Predictions of the presence of signal peptides in proteins that have reduced abundance at plasma membrane following ES2 treatment.

Supplementary Table S6. Prediction of transmembrane helices present in proteins that have reduced abundance at plasma membrane following ES2 treatment using DeepTMHMM.

(Supplementary Tables S1–S6 were attached as separate excel files.)

Supplementary Figure S1.

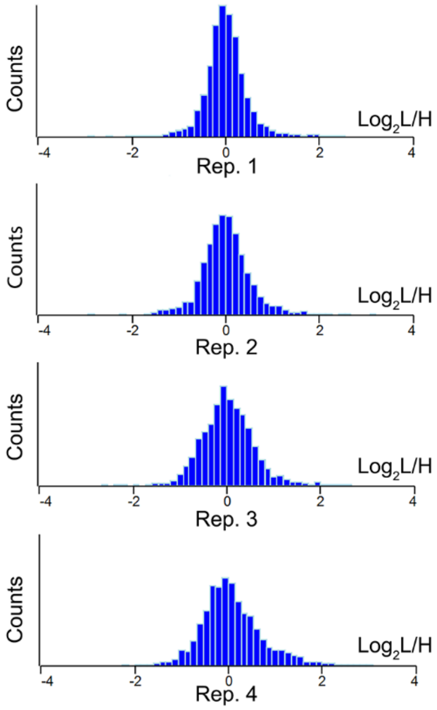

Supplementary Figure S1. Histogram of Log_2L/H distribution after median subtraction.

Supplementary Figure S2.

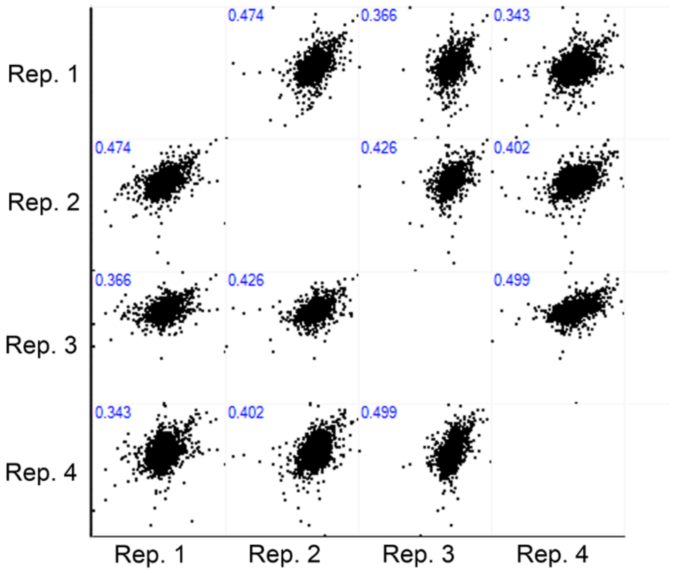

Supplementary Figure S2. Multi scatter plots of correlation coefficient between different replicates.

Supplementary Figure S3

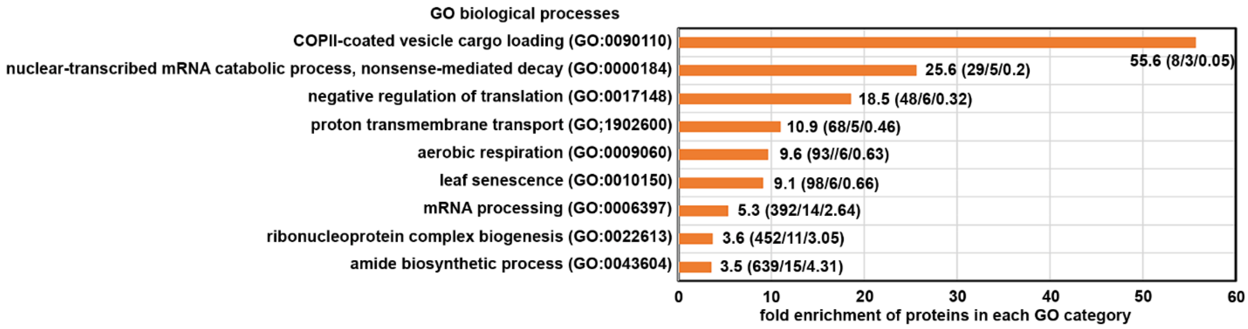

Supplementary Figure S3. Gene Ontology terms of biological processes category that are enriched in our protein list with significantly increased abundance after ES2 treatment.

Numbers on each row represent: fold enrichment (total number of proteins in the category/number of proteins identified in the reduced protein list/number of proteins expected in reduced protein list). Fold enrichment represents the ratio of the number of proteins identified divided by the number of proteins expected in the list.
